# Correction: Deletion of the Highly Conserved *N*-Glycan at Asn260 of HIV-1 gp120 Affects Folding and Lysosomal Degradation of gp120, and Results in Loss of Viral Infectivity

**DOI:** 10.1371/journal.pone.0110202

**Published:** 2014-10-03

**Authors:** The PLOS ONE Staff

## Notice of Republication

This article was republished on September 1, 2014, to correct errors in the references and in the legend for Figure 10 that were introduced during the typesetting process. Please download this article again to view the correct version. The originally published, uncorrected article and the republished, corrected article are provided here for reference.

## Supporting Information

Files S1 Originally published, uncorrected article.
(PDF)

Files S2 Republished corrected article.
(PDF)
