# Supplementary material for: Correction: Deletion of the Highly Conserved N-Glycan at Asn260 of HIV-1 gp120 Affects Folding and Lysosomal Degradation of gp120, and Results in Loss of Viral Infectivity
Source: PLoS One. 2014 Oct 3;9(10):e110202. doi: 10.1371/journal.pone.0110202 (PMC4184895; doi:10.1371/journal.pone.0110202)
Supplement: Files S1 — Originally published, uncorrected article. (PDF) [file pone.0110202.s001.pdf]

PLOS | ONE

Deletion of the Highly Conserved *N*-Glycan at Asn260 of HIV-1 gp120 Affects Folding and Lysosomal Degradation of gp120, and Results in Loss of Viral Infectivity

Leen Mathys[1]☯, Katrien O. François[1]☯, Matthias Quandte[2], Ineke Braakman[2], Jan Balzarini[1]*

1 Rega Institute for Medical Research, KU Leuven, Leuven, Belgium, **2** Cellular Protein Chemistry, Bijvoet Center for Biomolecular Research, Faculty of Science, Utrecht University, Utrecht, The Netherlands

Abstract

N-linked glycans covering the surface of the HIV-1 glycoprotein gp120 are of major importance for the correct folding of this glycoprotein. Of the, on average, 24 *N*-linked glycans present on gp120, the glycan at Asn260 was reported to be essential for the correct expression of gp120 and gp41 in the virus particle and deletion of the N260 glycan in gp120 heavily compromised virus infectivity. We show here that gp160 containing the N260Q mutation reaches the Golgi apparatus during biosynthesis. Using pulse-chase experiments with [^{35}S] methionine/cysteine, we show that oxidative folding was slightly delayed in case of mutant N260Q gp160 and that CD4 binding was markedly compromised compared to wild-type gp160. In the search of compensatory mutations, we found a mutation in the V1/V2 loop of gp120 (S128N) that could partially restore the infectivity of mutant N260Q gp120 virus. However, the mutation S128N did not enhance any of the above-mentioned processes so its underlying compensatory mechanism must be a conformational effect that does not affect CD4 binding per se. Finally, we show that mutant N260Q gp160 was cleaved to gp120 and gp41 to a much lower extent than wild-type gp160, and that it was subject of lysosomal degradation to a higher extent than wild-type gp160 showing a prominent role of this process in the breakdown of N260-glycan-deleted gp160, which could not be counteracted by the S128N mutation. Moreover, at least part of the wild-type or mutant gp160 that is normally targeted for lysosomal degradation reached a conformation that enabled CD4 binding.

Citation: Mathys L, François KO, Quandte M, Braakman I, Balzarini J (2014) Deletion of the Highly Conserved *N*-Glycan at Asn260 of HIV-1 gp120 Affects Folding and Lysosomal Degradation of gp120, and Results in Loss of Viral Infectivity. PLOS ONE 9(6): e101181. doi:10.1371/journal.pone.0101181

Editor: William A. Paxton, Institute of Infection and Global Health, United Kingdom

Received April 28, 2014; **Accepted** June 3, 2014; **Published** June 26, 2014

Copyright: © 2014 Mathys et al. This is an open-access article distributed under the terms of the Creative Commons Attribution License, which permits unrestricted use, distribution, and reproduction in any medium, provided the original author and source are credited.

Data Availability: The authors confirm that all data underlying the findings are fully available without restriction. All data are included within the manuscript.

Funding: This work was supported by grants from the KU Leuven Centers of Excellence (PF-10/018), the KU Leuven Geconcerteerde Onderzoeksacties (GOA) (no. 10/014), the Fonds voor Wetenschappelijk Onderzoek (FWO) (G.0485.08) and the CHAARM Network Project of the European Commission. Leen Mathys is supported by a fellowship from L'Oréal-UNESCO, in collaboration with the Belgian FWO (Aspirant). Matthias Quandte was supported by the European Union 7th framework program, ITN "Virus entry". The funders had no role in study design, data collection and analysis, decision to publish, or preparation of the manuscript.

Competing Interests: The authors have declared that no competing interests exist.

* Email: jan.balzarini@rega.kuleuven.be

☯ These authors contributed equally to this work.

Introduction

The envelope glycoprotein of the human immunodeficiency virus (HIV), gp160, consists of surface subunit gp120 and transmembrane subunit gp41. Gp120 is densely covered by *N*-linked glycans. This glycan shield protects the virus by hiding immunogenic epitopes, thus preventing efficient neutralization by the immune system. Several studies have indicated that the creation of "holes" in the glycan shield by removal of as few as two glycans, can lead to the production of new neutralizing antibodies against the previously hidden epitopes [1–3]. Therefore, the maintenance of an intact glycan shield is of utmost importance for the survival of HIV in the host.

In eukaryotes, protein *N*-glycosylation takes place in the endoplasmic reticulum (ER), resulting in high-mannose-type *N*-linked glycans covalently coupled to the nascent protein. In the Golgi apparatus, which contains a variety of glycosidases and glycosyltransferases, *O*-linked glycans are added and the high-mannose type *N*-glycans are processed. It is currently believed that high-mannose-type *N*-glycans promote protein folding in the ER and that the extensive processing of these glycans into complex-type glycans in the Golgi apparatus allows the further stabilization of functional protein conformations [4]. However, not all high-mannose-type *N*-glycans on the HIV envelope gp120 are processed to complex-type glycans. In particular, in many viral envelope glycoproteins, a significant percentage of *N*-glycans remains high-mannose-type.

The nature of the *N*-glycans on HIV gp120 is still a matter of debate. Several studies, performed on both recombinant gp120 or gp120 derived from persistently-infected human CD4^{+} T cells, suggested that at least half of the *N*-glycans present on the surface of the HIV envelope are complex-type oligosaccharides [5,6]. However, other studies have indicated that the glycans on HIV gp120 are predominantly high-mannose-type [7,8], but that the glycan distribution changes in favour of complex-type glycans when gp120 is recombinantly produced. In addition, when studying gp120 derived from pseudoviral production systems or infectious molecular clones, such as pLAI-JRCSF, the expression level of gp120 seems to influence the glycosylation profile: the

more envelope is expressed, the higher the high-mannose-type *N*-glycan abundance [7].

Several glycans are very important for viral infectivity. It has been reported that the glycan at amino acid position N260 is indispensable for efficient HIV infection of its target cells. This finding is valid for multiple HIV-1 strains including clade B [9–12] and clade BC [12]. This *N*-glycan has earlier been determined to be high-mannose-type when gp120 is expressed in Chinese hamster ovary cells [6]. We have recently shown that mutations in the *N*-glycosylation motif 260NGS262 result in an almost complete annihilation of gp120/gp41 incorporation in the virus particle envelope [13]. Mutations destroying this particular glycosylation motif in gp120 impede proper trafficking and subsequent incorporation of the envelope glycoprotein to the plasma membrane. In this study, we further investigated the molecular mechanisms behind this improper incorporation of the mutant gp120 in the viral envelope.

Materials and Methods

Cells

Human T lymphocytic C8166 cells were obtained from the American Type Culture Collection (Manassas, VA) and were cultivated in RPMI-1640 medium (Invitrogen, Merelbeke, Belgium) supplemented with 10% fetal calf serum (FCS) (Sigma, Bornem, Belgium), 1% streptomycin, 2 mM L-glutamine and 75 mM $NaHCO_3$. HEK293T cells were purchased from the American Type Culture Collection and cultivated in Dulbecco's Modified Eagle Medium (Invitrogen) supplemented with 10% FCS, 1% streptomycin and 75 mM $NaHCO_3$. HeLa cells were grown in MEM (Invitrogen) containing 10% FCS, 1% glutamax, 1% Pen/Strep and 1% non-essential amino acids.

Plasmids

The pNL4.3-Δenv-EGFP construct was used for production of wild-type (WT) NL4.3 virus after recombination with *env*. For this molecular clone, the expression of enhanced green fluorescent protein (EGFP) in infected cells is a measurement of virus production as described previously [14]. The construct pNL4.3-ΔEnv-EGFP was a kind gift from Dr. M.E. Quiñones-Mateu (Lerner Research Institute).

The construct pNL4.3ΔGagΔPol_EGFP was derived from the vector pNL4.3ΔGagPr_EGFP, which was described previously [15]. The *pol* gene was removed by inverse PCR using the Herculase II DNA polymerase (Agilent Technologies, Diegem, Belgium). The primers 5′-CGACGCTCTCGCACC-CATCTCTG-3′ (position 785–807 in HIV-1 NL4.3 [Genbank accession number M19921]) and 5′-CACARGGAAAAGAT-TAGTAAAACACCATATGTATATTTC-3′ (position 5106–5135) were used to delete the entire *pol* gene and were a kind gift from Prof. K. Van Laethem. The parental DNA was digested with DpnI (Fermentas, Leuven, Belgium) and the vector was self-ligated using the Quick Ligation kit (New England Biolabs, Leiden, The Netherlands). Correct removal of the *pol* gene and ligation of the vector was confirmed by restriction enzyme digestion of the vector and vector sequencing.

For transient transfection of WT and mutant gp160 in pulse-chase analysis we constructed a plasmid containing a CMV promoter and IntronA [16], followed by the HIV-1 LAI gp160 sequence. The plasmid, which we called pMQ, allows high transient expression of gp160 without codon optimization.

Site-directed mutagenesis of *gp120*

The plasmid pBlue-env which encodes the *env* gene [17,18] was used to generate gp120 mutant virus strains with mutations at amino acid positions 260 (N to Q), 120 (V to A), 128 (S to N) and 302 (R to I). These mutations were introduced into pBlue-env using the Quikchange Site-Directed Mutagenesis Kit (Agilent Technologies). Plasmid DNA was purified by the PureLink Quick Plasmid Miniprep Kit (Invitrogen). The presence of the mutations was confirmed by sequencing the *gp120* gene as described previously [19].

Mutation N260Q, alone or in combination with mutation S128N in gp120 were also introduced in vectors pNL4.3ΔGagΔ-Pol_EGFP and pMQ, using the same strategy as described above.

Generation of mutant virus by env chimeric virus technology

The generation of mutant virus was performed as described previously [20]. Briefly, HEK293T cells were transfected with 10 μg of linearized pNL4.3-ΔEnv-EGFP and 2 μg of isolated PCR product of the WT or mutant HIV-1 envelope glycoprotein, using the calcium chloride method. Three days post transfection, the recombinant virus and/or the transfected 293T cells were harvested.

To study the effect of lysosomal inhibitors on the processing of the viral envelope, 20 μM chloroquine was added to the 293T cells 30 minutes before the addition of viral DNA.

Virus evolution

Evolution experiments were adapted from experiments described before [21,22]. Briefly, 1.10^6 C8166 cells were transfected using the Amaxa Nucleofector Technology (Lonza, Verviers, Belgium) with 10 μg of the plasmid pNL4.3-ΔEnv-EGFP, together with 2 μg of isolated PCR product of the envelope of HIV-1, containing the N260Q mutation. The spread of virus was monitored by fluorescence microscopy, based on the expression of EGFP. Medium was harvested at regular time points and added to uninfected cells. When infection was established, the virus was harvested and sequenced as described before [19].

Infectivity of WT and mutant virus strains

To determine the infectivity of the virus strains with compensatory mutations in gp120, different amounts of virus (5,000–2,500–1,250–625–312.5 pg p24), harvested from transfected 293T cells, were added to 30.000 C8166 cells in a total volume of 200 μl. Three days post infection, the cells were fixed with 3% paraformaldehyde and infection was monitored with the FACS-CantoII flow cytometer (BD biosciences, Erembodegem, Belgium).

Expression of gp160, gp120 and gp41 in the virus particle and in the transfected 293T cell

The expression levels of the envelope proteins gp160, gp120 and gp41 in both virus particles and transfected 293T cells were determined by western blot (WB) analysis as described before [13]. In the case of WB on virus lysates, 50 ng of p24 was loaded onto a 4–12% Bis-Tris PAGE gel (Invitrogen), while for cell lysates 2 ng of p24 was loaded.

Cell lysates were also subjected to endoglycosidase H (EndoH) digestion (New England Biolabs), according to the manufacturer's protocol. An amount of 2 ng of p24, present in the cell lysates, was denatured with the glycoprotein-denaturing buffer supplied by the manufacturer. After a 10-minute incubation at 99°C, samples were cooled down and G5 reaction buffer was added. Finally, 1 μl of EndoH, corresponding to 500 U of the enzyme, was added, and

samples were incubated at 37°C for at least 3 hours. Before loading the samples onto a 4–12% Bis-Tris gel, 4x LDS sample buffer (Invitrogen) was added.

Finally, complete deglycosylation of the cell lysates was obtained through peptide-N-glycosidase F (PNGaseF) digestion (Roche, Vilvoorde, Belgium). The samples (2 ng of p24) were boiled for 10 minutes in a denaturation buffer (10 mM sodium phosphate pH 7, 1% SDS). After addition of the incubation buffer (10 mM sodium phosphate pH 7, 1% Triton X-100, 1 mM EDTA, 0.1% β-mercaptoethanol) and 1 μl of PNGaseF, samples were incubated for at least 3 hours at 37°C. Before loading the samples onto the 4–12% Bis-Tris gel, 4x LDS sample buffer was added.

Quantification of the WB data was performed using the ImageJ software.

MagicMark XP Western Protein Standard (Novex, Merelbeke, Belgium) was used to estimate the molecular weight of the proteins.

Intracellular staining of the ER and Golgi in HIV-transfected 293T cells

For intracellular staining experiments, 293T cells were seeded in 8-well chamber slides (Ibidi, Beloeil, QC) and transfected with pNL4.3ΔGagΔPol_EGFP WT or N260Q gp120 constructs as described above. Two to three days after transfection, the cells were carefully washed with PBS, and fixed in 4% paraformaldehyde. Permeabilisation was done with 0.1% Triton X-100, after which 10% normal goat serum (Invitrogen) was added for blocking. Gp120 was detected in the transfected 293T cells using antibody 2G12 (Polynum, Vienna, Austria). For ER and Golgi staining, antibodies against PDI and giantin were used, respectively. Corresponding secondary antibodies were either labelled with Alexa633 (for 2G12) or Alexa568 (for the organelles) (all purchased from Invitrogen). Images were taken using a laser scanning SP5 confocal microscope (Leica, Diegem, Belgium).

Pulse-chase experiments

Twenty-four hours in advance, HeLa cells were transfected with a mix of polyethylenimine (pEI) and WT or mutant DNA in a ratio of 2.5 to 1, after which the cells were subjected to pulse-chase analysis as described before [23]. In short, cells were starved in medium lacking cysteine and methionine for 15–30 min and pulse labelled for 10 min with 55 μCi/35 mm dish of Express ^{35}S protein labelling mix (PerkinElmer, Boston, MA). The pulse was stopped and the chase started by the first of 2 washes with chase medium containing an excess of cold cysteine and methionine. At the end of each chase time, cells were cooled on ice and further disulfide bond formation and isomerization was blocked with 20 mM iodoacetamide (IAM).

Cells were lysed and detergent lysates were subjected to immunoprecipitation with polyclonal antibody 40336 against gp160 or with CD4-IgG2 against the CD4-binding site. Next, samples were deglycosylated using EndoH (Roche) as described above, and were subjected to non-reducing and reducing (25 mM DTT) 7.5% SDS-PAGE [23,24]. Gels were dried and exposed to Kodak MR films for autoradiography.

Flow cytometric analysis of gp120 expression on the surface of HIV-transfected 293T cells

293T cells were transfected with linearized pNL4.3-ΔEnv-EGFP and isolated WT or mutant gp160 PCR product using the calcium chloride method, in the absence or presence of 20 μM chloroquine. Two or three days post transfection, the cells were stained to detect surface gp120 using the monoclonal antibody 2G12 and a secundary antibody labelled with Alexa Fluor 647 (Invitrogen). The cells were analysed using the FACSCanto II flow cytometer (BD Biosciences).

Quantification of soluble CD4 (sCD4) binding using an enzyme-linked immunosorbent assay (ELISA)

This experiment was performed as described previously [13], with some adaptations. 8-well maxisorp strips (Nunc, Erembodegem, Belgium) were coated with 0.5 μg of sCD4 (Sino Biological Inc, Zoersel, Belgium) in 50 mM carbonate buffer (pH 9.6) during 2 hours at room temperature. Then, the wells were blocked for 1 hour at room temperature using PBS pH 7.4 supplemented with 0.05% Tween 20 (Sigma) and 2% milk powder (= blocking buffer). After washing the wells with wash buffer (PBS supplemented with 0.05% Tween 20), 45 ng p24 of virus lysed using 10% Triton-X100 in blocking buffer was added to the wells for 1 hour at 37°C. After washing, the wells were incubated with the primary sheep antibody directed against gp120 (D7324; Aalto Bio Reagents, Dublin, Ireland) in blocking buffer during 1 hour at 37°C. Afterwards, the wells were washed again with wash buffer and were incubated for 1 hour at 37°C with the secondary antibody labelled with alkaline phosphatase in blocking buffer. Substrate buffer (1 mg/ml p-nitrophenyl phosphate (Sigma) dissolved in 10% diethanolamine, pH 9.8, with 0.5 $MgCl_2$) was added to the wells after sufficient washing. Following 30 minutes incubation, the absorbance at 405 nm as a measurement of virus binding to CD4 was determined using the Safire 2 microtiter plate reader (Tecan, Mechelen, Belgium).

Results

Mutated N260Q gp160 proceeds from the endoplasmic reticulum to the Golgi

We have previously shown that in HIV-transfected 293T cells, gp160 containing the N260Q mutation appeared in the Golgi for further processing [13]. To confirm the efficient transport of gp160 to the Golgi, we performed intracellular staining of 293T cells transfected with WT or mutant gp160. Figure 1 shows the co-localization of gp160 with markers for the ER (PDI) and the Golgi apparatus (giantin). A large fraction of both WT and mutant N260Q gp160 resided in the ER, which is the organelle in which protein biosynthesis takes place. In steady state, most of the gp160 shows ER localization due to its slow folding kinetics [23]. Co-staining was also seen in the Golgi apparatus for both WT and mutant N260Q gp160 (Fig. 1). These results indicated that (WT and mutant) gp160 is continuously produced in the ER, but also that (at least a fraction of) the mutant envelope proceeded to the Golgi.

An important step during the processing of gp160 is the conversion of part of the high-mannose-type *N*-glycans (added to asparagines during polypeptide synthesis) into complex-type *N*-glycans. This conversion takes place in the Golgi where addition of an N-Acetylglucosamine (GlcNAc) residue to the glycan represents the first step in the conversion to a complex-type glycan [25]. To confirm that mutant N260Q gp160 is processed in the Golgi, we treated cell lysates of transfected 293T cells with EndoH, which only cleaves high-mannose-type and hybrid-type glycans, but not complex-type glycans. As can be seen from the Western blot analysis in Figure 2, Panel A, WT and mutant gp160 were EndoH sensitive, indicating that the majority of WT and mutant N260Q gp160 had high-mannose-type glycans and resided in the ER.

The same samples were also treated with PNGaseF, which cleaves off all *N*-linked glycans and converts the asparagines (lacking the glycan) into aspartic acid residues. This digestion

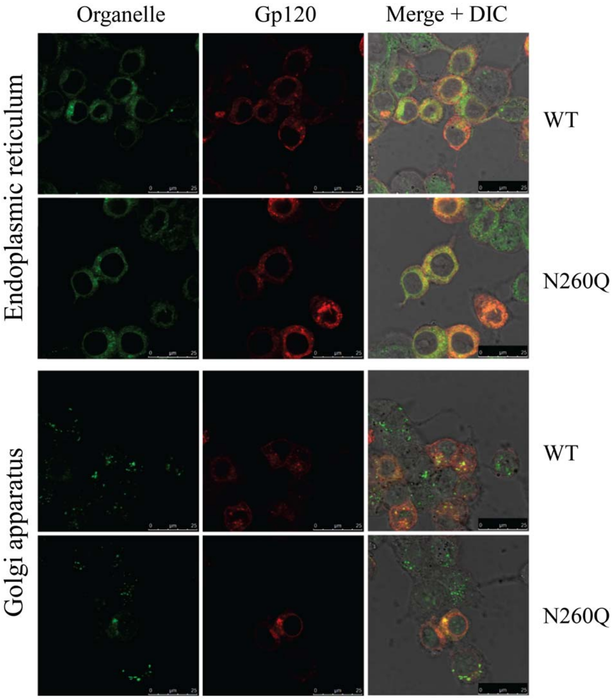

Figure 1. Intracellular staining of HIV-transfected HEK293T cells. The ER and Golgi apparatus (green) were labelled with an anti-PDI and an anti-giantin antibody, respectively. Gp120 (red) was visualized with antibody 2G12. Overlay of gp120 and the ER or Golgi is shown as orange/yellow.
doi:10.1371/journal.pone.0101181.g001

resulted for both WT and mutant gp160 in a major band with a slightly lower molecular weight than the EndoH-treated samples because EndoH leaves 1 GlcNAc attached to the asparagine, which is cleaved off by PNGaseF digestion. These results indicate that both EndoH and PNGaseF digested gp160 are deglycosylated (Fig. 2, Panel A), indicating that the vast majority of both WT and mutant N260Q gp160 were not yet processed in the Golgi apparatus. Whereas Fig. 2A shows a Western blot analysis of cell lysates exposed to an anti-gp41 antibody, Fig. 2B shows an identical Western blot analysis of cell lysates exposed to an anti-gp120 antibody. This explains why in the non-treated lysates, a double band was observed that corresponds to glycosylated gp160 and gp120. The lower band (approximately 90–95 kDa) seen in both Fig. 2A and 2B represents most likely untranslocated gp160 that did not get glycosylated and still had its signal peptide. The predominant single upper band that appeared in the mutant cell lysates (instead of a double band in the WT cell lysates) is suggestive for the presence of mainly uncleaved gp160. This indicates that the mutant envelope gp160 is less efficiently cleaved to gp120+gp41 than WT gp160.

Mutation in the V1/V2-loop of gp120 can only moderately compensate for the loss of infectivity due to the N260Q gp120 mutation

Previous attempts by site-directed mutagenesis to introduce new glycosylation sites in the proximity of the 260NGS262 glycosylation motif failed to restore the infectivity of the N260Q gp120 mutant virus [13]. However, continuous passaging of seemingly uninfectious virus in cell culture has been shown earlier to result in the selection of compensating mutations [26,27]. We therefore investigated whether the detrimental loss of infectivity due to the N260Q mutation in gp160 could be compensated for by the introduction of a second-site suppressor mutation in gp160. For this purpose, C8166 cells were transfected with the HIV-1 construct encoding mutant N260Q gp160. After long-term passaging of these transfected C8166 cells, two mutations occurred in the V1/V2-loop of gp120 that allowed cell-free infection (albeit still poor) of mutant HIV containing N260Q gp160. The mutation at amino acid position 120, changing a valine into an alanine, appeared first during the virus evolution assays as an addition to the pre-existing N260Q gp120 mutation. Later on, the S128N mutation appeared in combination with V120A and N260Q. At the end of the selection process, solely the S128N and N260Q gp120 mutations were retained in gp160. The N260Q mutation in gp120 apparently did not revert to WT.

To determine to what extent infectivity was restored by the V1/V2-mutations, we introduced the single V120A and S128N mutations and the combined V120A/S128N mutations into the mutant N260Q gp160 background. Figure 3 shows that the V120A and V120A/S128N gp120 mutations were virtually unable to restore infectivity of mutant N260Q gp160 HIV-1. On the other hand, the single S128N mutation in the N260Q gp160 background allowed about 7% infection at the highest dose of virus added to the uninfected C8166 cell cultures (Fig. 3), thus being still more than 10 times less infectious than WT virus.

Despite long-term passaging (>30 passages) of the mutant N260Q/S128N gp120 virus, mutations that further increased viral infectivity did not arise. It has been reported earlier that an arginine to isoleucine mutation at amino acid position 302 in gp160 restored infection potential of the mutant N260Q/S128N virus [28]. Although this mutation was not selected in our experiments, we aimed to confirm these observations by introducing the R302I mutation in the HIV-1 gp160 that already contained the N260Q, N260Q/V120A, N260Q/V120A/S128N and N260Q/S128N background mutations. Different concentrations of these mutant viruses were added to C8166 cell cultures, and the degree of infection was determined based on the appearance of EGFP by FACS analysis. The R302I mutation on its own did not alter the infectivity of the virus. In contrast to the report by Willey *et al.* [28] none of the viruses that contained the R302I mutation in combination with the N260Q or N260Q/S128N gp120 mutations showed any infectivity gain (data not shown). Thus, we identified S128N in the V1/V2-loop as a compensatory mutation to N260Q (Fig. 4), which restores mutant virus infectivity up to <10% of WT levels.

The S128N compensatory mutation in gp120 has no measurable effect on the gp120/gp41 levels in mutant virus particles

We have previously shown that the N260Q mutation in gp160 of HIV-1 is associated with dramatically lower levels of gp120 and gp41 expression in the virus particle [13]. Since the S128N mutation partially restored viral infectivity, we wondered whether this was caused by increased incorporation of gp120 and gp41 in the virus particle. To test this hypothesis, the virus in the cell culture medium of transfected 293T cells was concentrated, lysed, and subjected to WB analysis. Although the gp120 and gp41 levels in mutant N260Q gp120 virus were somewhat higher in this experiment than previously reported, its levels were still markedly lower than WT levels (Fig. 5A, 5B, 5C: data without chloroquine). Introduction of the S128N mutation in the mutant N260Q gp160 virus did not increase the expression and incorporation of both gp120 and gp41 in the virus particle.

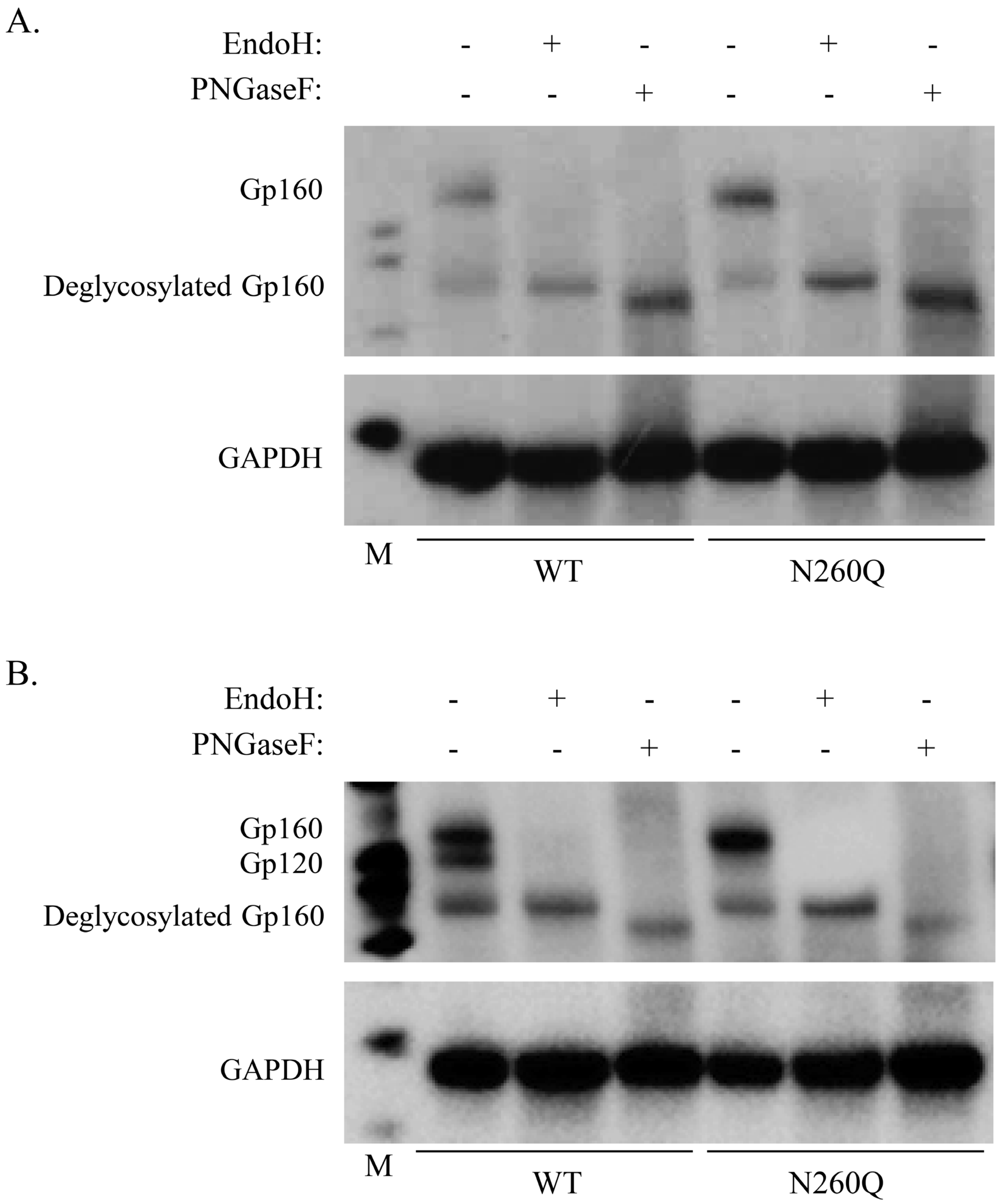

Figure 2. Western blot analysis of glycosidase-treated cell lysates derived from wild-type (WT) and mutant N260Q gp160 HIV-transfected HEK293T cells. Cell lysates were treated with either endoglycosidase H (EndoH) or peptide-N-glycosidase F (PNGaseF). The glycoprotein gp160 was detected with an anti-gp41 antibody (A) or an anti-gp120 antibody (B). GAPDH was used as an equal-sample loading control. M: MagicMark XP Western Protein Standard (Novex).
doi:10.1371/journal.pone.0101181.g002

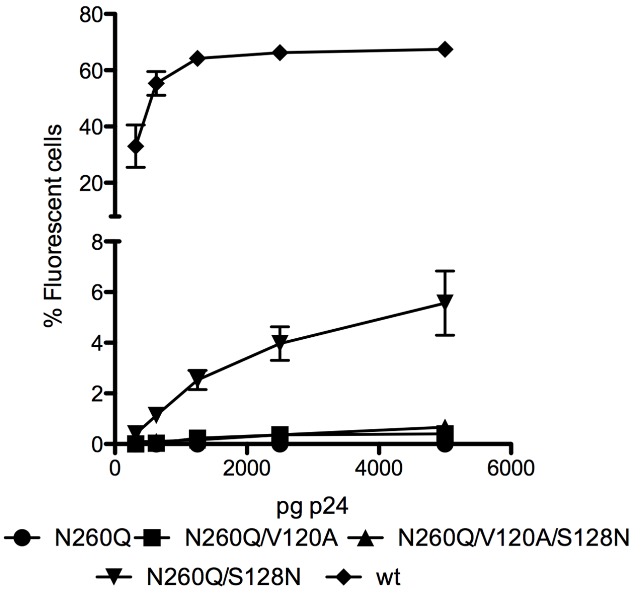

Figure 3. Infectivity of WT and mutant virus strains. Different concentrations of (WT and mutant) virus were added to C8166 cell cultures. Three to four days post infection, the viral infection was quantified by FACS analysis. Data (± SD) are the means of 2 to 3 independent experiments.
doi:10.1371/journal.pone.0101181.g003

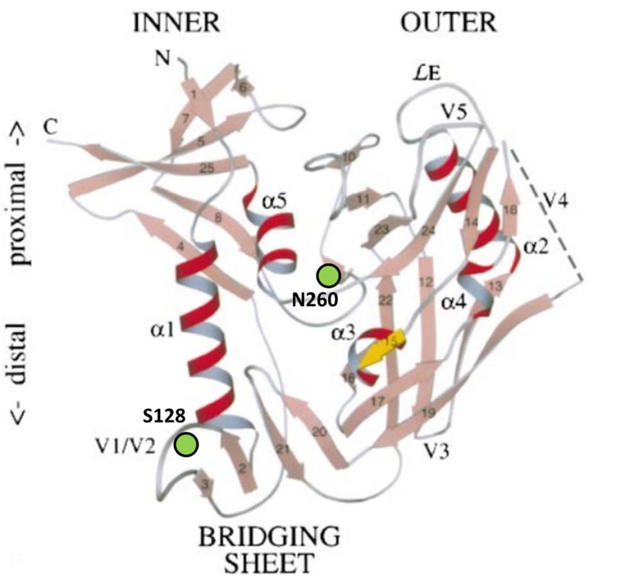

Figure 4. Ribbon diagram representation of core gp120 with indication of Asn260 and Ser128. In the shown orientation, the viral envelope would be on top of the picture and the cellular target membrane on the bottom of the picture. Asn260 is positioned in the C2 region. Ser128 can be found at the base of the V1/V2 loop region. Amino acid numbering is based on HIV-1 strain NL4.3, and corresponds with amino acids Asn262 and Ser128 in HIV-1 strain HXB2. Figure adopted from Kwong *et al.* 1998 [36].
doi:10.1371/journal.pone.0101181.g004

Maturation of mutant N260Q gp160 and mutant N260Q/S128N gp160 is delayed *versus* WT gp160

We next performed pulse-chase experiments with WT, N260Q gp160 and N260Q/S128N gp160 to study the effect of these mutations on the kinetics and yields of oxidative folding in the ER. During a short pulse-labelling with [^{35}S] methionine and [^{35}S] cysteine, all newly synthesized proteins were radioactively labelled. This cohort of proteins was subsequently followed over time, allowing us to monitor gp160's oxidative folding, signal peptide cleavage and gp120 shedding. After immunoprecipitation of detergent cell lysates with polyclonal anti-gp160 antibody and deglycosylation, gp160 samples were subjected to non-reducing and reducing SDS-PAGE.

As reported earlier, gp160's signal peptide is cleaved post-translationally [23,29]. On a reducing gel deglycosylated gp160 with its signal peptide still attached, had an apparent molecular weight of ~100 kDa (Fig. 6A). After ~15 min, a second band with increased mobility appeared representing signal peptide-cleaved gp160. Over time, virtually all gp160 lost its signal peptide. Gradually, the signal intensity decreased, which was due to a decrease in cell-associated gp120 since cleavage of the signal sequence results in env trimerization, transport to the Golgi and subsequently to the plasma membrane where gp120 sheds from gp41. This is demonstrated in Figure 6C, where gp120 in the supernatant is shown.

On a non-reducing gel the increasing number of disulfide bonds formed during folding increased compactness and hence mobility of gp160 (Fig. 6B). Immediately after synthesis, gp160 ran as a smear, representing folding intermediates with incomplete or non-native sets of disulfide bonds. The folding intermediates moved into a single band as disulfide bond formation and isomerization continued. This single band represented native, completely folded gp160.

Figure 6B, showed slightly delayed maturation of both N260Q and N260Q/S128N gp160. While signal peptide cleavage seemed similar to WT gp160 (Fig. 6A), fewer molecules formed native gp160 and cell surface arrival and shedding were delayed (Fig. 6C). The delay caused by N260Q was not diminished by the addition of S128N. The compensatory mutation hence did not markedly affect oxidative folding of N260Q gp160.

Only a minority of ER-lumenal mutant N260Q gp160 and N260Q/S128N gp160 is CD4-binding competent

We demonstrated earlier that mutant N260Q gp160 was CD4-binding compromised [13]. Since the compensatory mutation S128N did not improve overall oxidative folding of gp160 we wondered whether it affected CD4 binding. Hence, we performed pulse-chase analyses as described above. The lysates were pulled-down with CD4-IgG2 and subjected to SDS-PAGE after deglycosylation. WT gp160 showed little recognition by CD4-IgG2 immediately after synthesis (Fig. 7, upper panel, lane of 0 min). The amount of bound gp160 increased over time. On a non-reducing gel mainly oxidized gp160 that ran in the position of native protein efficiently bound to CD4 (Fig. 7, lanes of 15 min up to 4 h). This is in agreement with the discontinuous nature of the CD4-binding epitope of gp160. Gp160 needs to fold to some degree to become CD4-binding competent. Mutant N260Q gp160 was recognized much less by CD4-IgG2 than WT gp160 (Fig. 7, middle panel). The signal intensity was strongly decreased while total quantity of gp160 was not different in the mutant (see Fig. 6). Hence, a fraction of mutant N260Q gp160 folded properly and became CD4-binding competent but the majority failed to do so.

The compensatory mutation S128N did not visibly improve recognition of mutant N260Q gp160 by CD4-IgG2 (Fig. 7, lower panel). We concluded that lack of the glycosylation site at position 260 severely compromised formation of the CD-binding site and the compensatory mutation in the V1/V2-loop (S128N) did not restore CD4-binding ability.

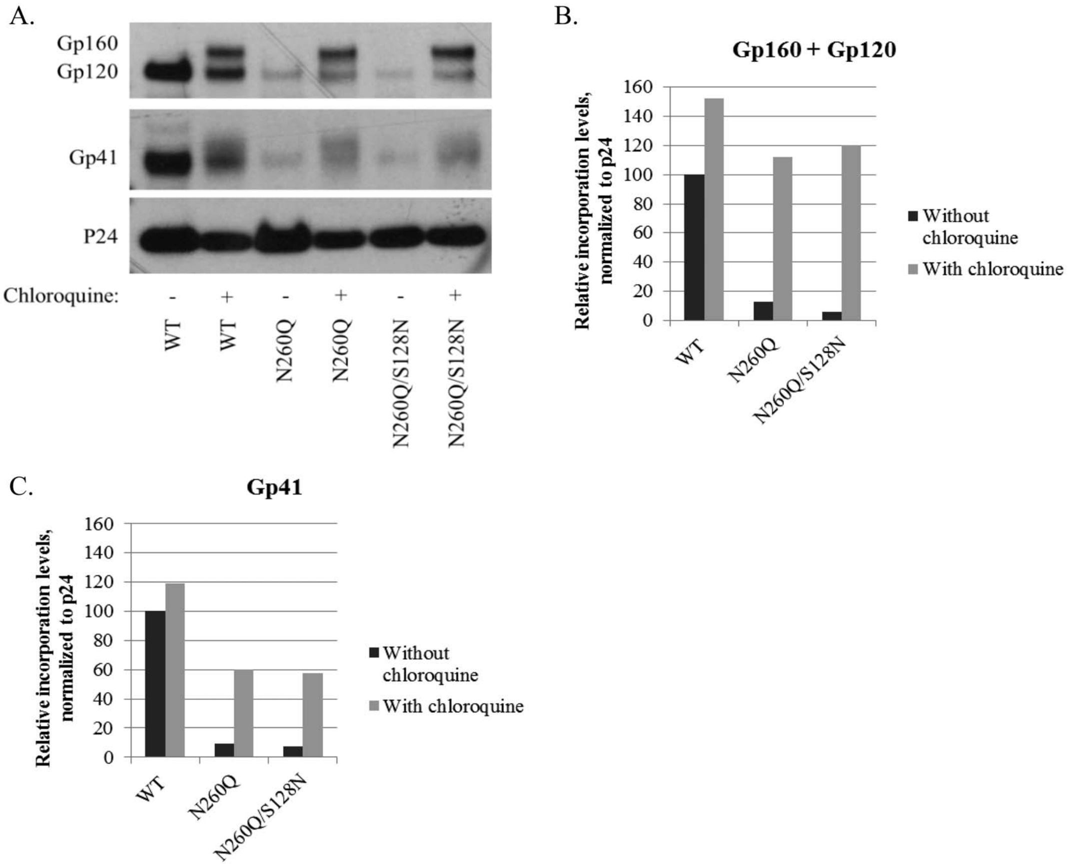

Figure 5. Western blot analysis of the incorporation levels of gp160, gp120 and gp41 in viral particles. (A) Analysis of the level of gp160, gp120 and gp41 incorporation in virus particles, produced in the absence or presence of the lysosomal inhibitor chloroquine. P24 was quantified as an equal loading control. (B–C) Relative protein levels as quantified based on panel A using the ImageJ software, and normalized to equal p24 levels.
doi:10.1371/journal.pone.0101181.g005

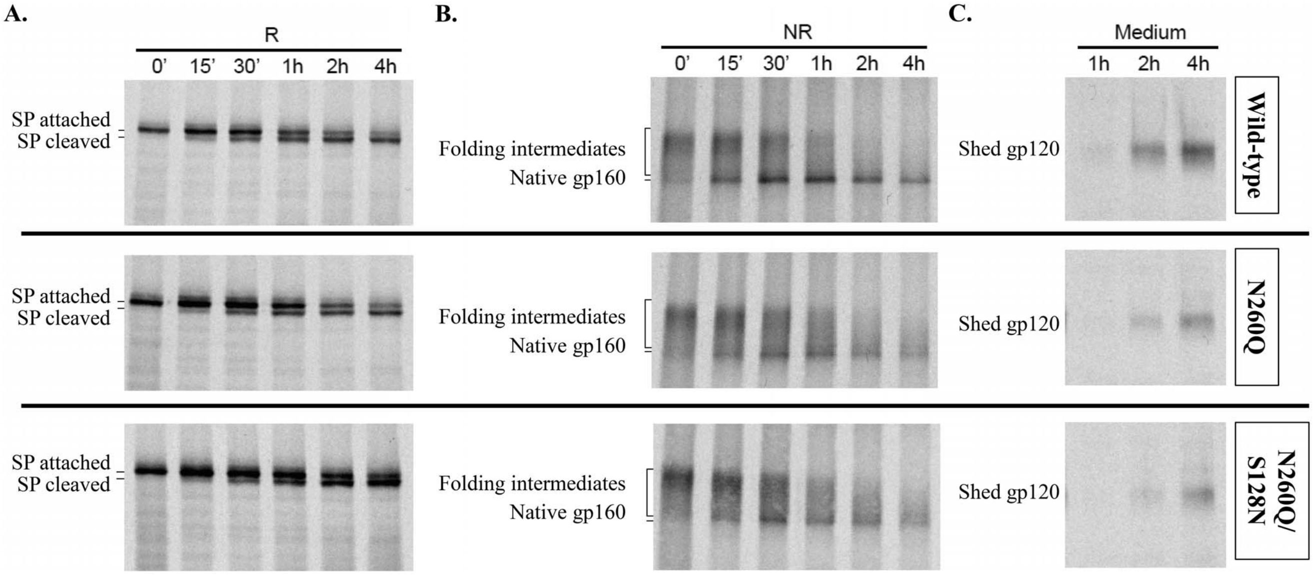

Figure 6. Pulse-chase experiments to study signal peptide cleavage, oxidative folding and gp120 shedding. HeLa cells transfected with WT or mutant gp160 were pulsed with Express ^{35}S protein labelling mix during 10 min and chased with cold cysteine and methionine for the indicated time. Films in panel A were exposed for 7 days and in panel B and C for 14 days. (A) Reducing (R) SDS-PAGE to study signal peptide cleavage. (B) Non-reducing (NR) SDS-PAGE to study oxidative folding. (C) Detection of shed gp120 in culture medium. SP: signal peptide.
doi:10.1371/journal.pone.0101181.g006

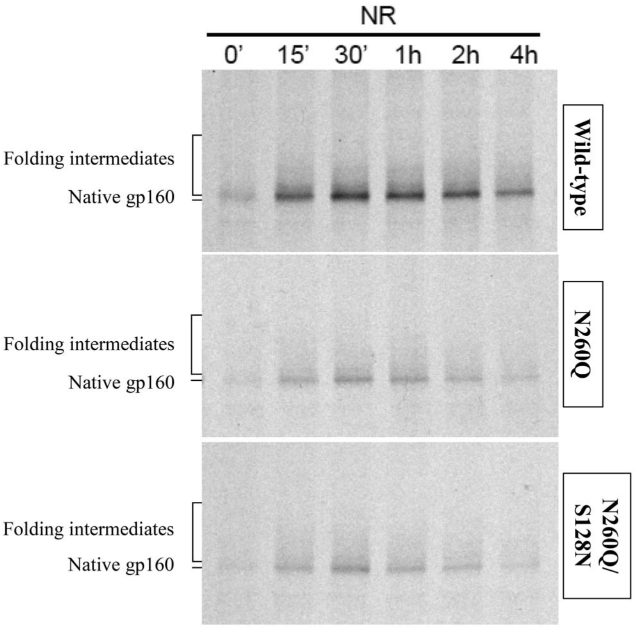

Figure 7. Immunoprecipitation of WT and mutant gp160 with CD4-IgG2. Aliquots of detergent lysates from Figure 6 were used for gp160 pull-down with CD4-IgG2, deglycosylation and subjection to SDS-PAGE. Films were exposed for 1 month.
doi:10.1371/journal.pone.0101181.g007

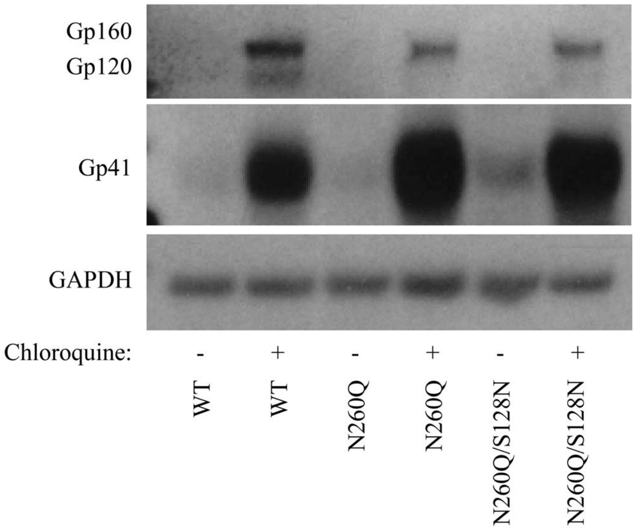

Figure 8. Western blot analysis of the expression levels of gp160, gp120 and gp41 in virus transfected HEK293T cells, in the absence or presence of the lysosomal inhibitor chloroquine. WT, N260Q and N260Q/S128N virus transfected HEK293T cells were lysed and subjected to WB analysis (2 ng of p24 protein), to detect gp160, gp120 and gp41 in the cell lysates. GAPDH was used as an equal loading control.
doi:10.1371/journal.pone.0101181.g008

The N260Q mutation increases lysosomal degradation of gp160, gp120 and gp41

Incorrectly folded proteins that do not pass the quality control in the ER are normally degraded by the proteasome after ubiquitination. However, certain proteins with minor conformational defects can pass the quality control of the ER and transit to the Golgi, where they are nonetheless detected as misfolded and diverted to lysosomes for degradation [30]. Given the fact that eventual expression of either glycoprotein on the viral envelope surface was low, we investigated the possibility that mutant N260Q gp120 and gp41 are degraded in lysosomes.

For this purpose, we added chloroquine to the 293T cells at the time of transfection, and the viruses derived from these cells, together with the cell lysates, were subjected to Western blot analysis. Chloroquine is known to block lysosomal activity. Figure 5A shows that in the case of WT virus, the addition of chloroquine to the 293T cells led to the incorporation of uncleaved gp160 in the virus particle. As shown previously, the N260Q mutation in gp120 is associated with a much lower incorporation of gp120 and gp41 in the virus particle envelope [13]. However, mutant N260Q gp120 virus derived from chloroquine-treated cells showed an increased level of gp120, gp41, and in particular gp160, in the virus particle (Fig. 5A, 5B, 5C). The relative increase of incorporated gp160, gp120 and gp41 was more pronounced for mutant N260Q gp120 HIV compared to WT HIV, suggesting that the N260Q mutation in gp160 promoted lysosomal degradation. The Western blot analysis in Fig. 5A for the mutant envelope confirms our findings (shown in Fig. 2B) that mutant gp160 is much less efficiently cleaved than wild-type gp160. Again, mutation S128N did not change the findings for N260Q gp160, whether with or without chloroquine.

WB analysis of the cell lysates of transfected 293T cells clearly showed that chloroquine treatment was accompanied with a massive increase in both gp160 and gp41 (Fig. 8).

To confirm the data obtained by WB analysis, HIV-transfected cells were stained with the monoclonal antibody 2G12 to detect gp120 on the cellular surface. Therefore, 293T cells were transfected with WT, N260Q or N260Q/S128N gp160-encoding HIV, in the absence or presence of chloroquine. It was shown that inhibition of lysosomal activity had a limited negative effect on the surface expression of WT gp120, while there was a marked increase in the level of surface gp120 in the case of the N260Q mutation (Fig. 9). Chloroquine also increased the level of N260Q/S128N gp120 on the cell surface, although less pronounced compared to the single mutant.

At least part of the gp160 that is targeted for lysosomal degradation is CD4-binding competent

We finally investigated the sCD4-binding potential of virions produced in the presence of chloroquine, to determine whether the conformation of the otherwise lysosomally degraded gp120 enables CD4 binding. Using an ELISA, it was confirmed that WT virus is able to bind CD4 substantially better than virus containing the gp120 N260Q or N260Q/S128N mutations (Fig. 10). The presence of chloroquine during virus production resulted for all viruses in an increase in CD4 binding potential, which was most pronounced for the N260Q gp120 mutant. This indicates that at least part of the otherwise degraded gp120 reached a conformation that supports CD4 binding.

Discussion

The *N*-glycan at amino acid N260 in HIV-1 gp120 is of utmost importance for proper infectivity of the virus against its target cells, such as $CD4^{+}$ T lymphocytes [13]. We have previously reported that mutations affecting the N260 glycosylation motif – either in the asparagine residue, or in the serine residue – severely impair incorporation of gp120 and gp41 in the mutant virus particles [13]. Here we show that, in steady state, gp160 is largely found in the ER because it folds slowly and takes a long time to exit the ER.

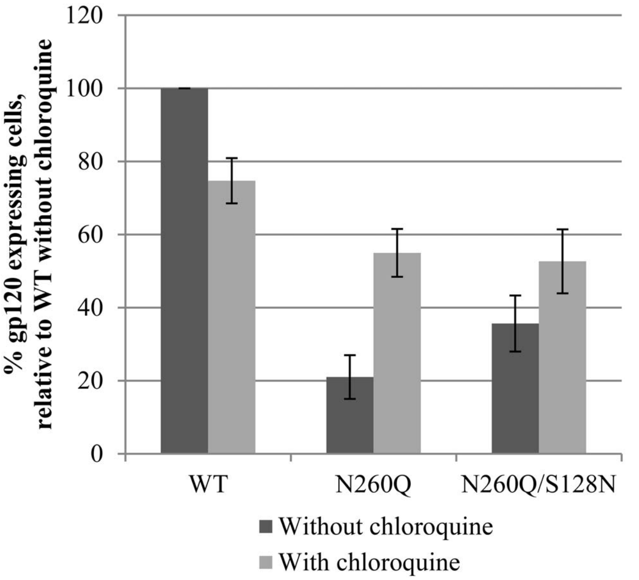

Figure 9. Flow cytometric analysis of gp120 expression on the surface of HIV-transfected HEK293T cells, in the absence or presence of chloroquine. Two days after transfection in the absence or presence of chloroquine to produce WT, N260Q or N260Q/S128N virus, HEK293T cells were stained to detect gp120 on the cell surface with the primary antibody 2G12 and a secondary anti-human antibody labelled with Alexa Fluor 647. Data are the means (± SEM) of 5 independent experiments.
doi:10.1371/journal.pone.0101181.g009

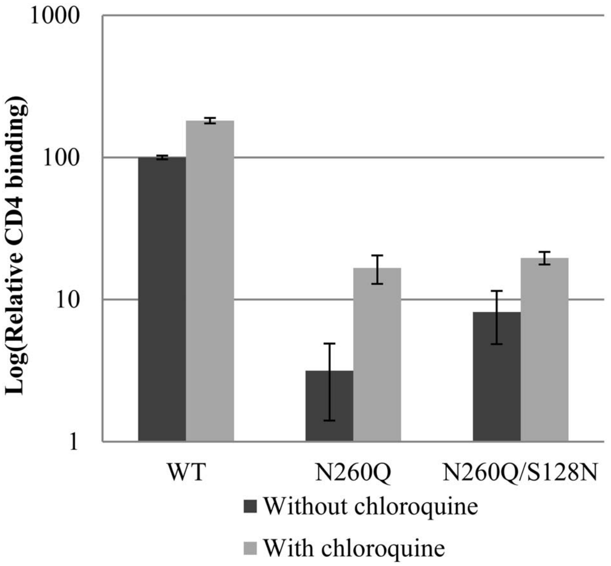

Figure 10. Capacity of WT and mutant virus strains, produced in the absence or presence of chloroquine, to bind to sCD4. 45 ng p24 of virus was lysed using Triton-X100 and was brought into contact with ELISA strips coated with sCD4. The binding efficiency was calculated as relative to the binding of WT virus produced in the absence of chloroquine. Data are the means (± SEM) of 2–3 independent experiments.
doi:10.1371/journal.pone.0101181.g010

These results correspond to what is reported in literature [23]. Additionally we show that mutant N260Q gp160 folds even slower than WT gp160. Although the infectivity of N260Q gp160 HIV could be somewhat increased by the introduction of the compensatory mutation S128N in gp160, this secondary mutation was found not to result in measurably increased expression/incorporation of the glycoprotein in the viral envelope, faster folding kinetics or improved CD4 binding. Hence, the underlying compensatory mechanism of the S128N mutation is currently not known.

Moore *et al.* [31] used antibodies to study the conformational changes induced by N260Q and its compensatory mutation S128N in gp160. In contrast to our results, they observed a complete restoration of CD4 binding upon introduction of S128N. The authors located mutation S128N in the C1 domain, and hence studied mostly the interaction of this domain with the C2 domain, which contains the N260 glycan. However, amino acid S128 is located in the V1/V2-loop, and the interaction of this loop with the C2 domain has not been investigated yet.

Willey *et al.* [28] reported the beneficial effect of the additional mutation at amino acid R302I in gp120 on mutant virus infectivity. However, even after long-term passaging of N260Q/S128N gp120-containing virus strains, we did not select for any additional mutation including R302I that could restore infection of the double mutant virus to WT levels. Also, in our hands, introduction of R302I in the N260Q/S128N gp120 background did not increase infectivity to WT levels. Although both our and Willey's studies were performed in the NL4.3 background, Willey *et al.* produced the mutant viruses in SW480 cells and used A3.01 cells for infectivity testing of the SW480 cell supernatants. We suggest that the choice of producer cells and/or target cells for infectivity testing is at the basis of this discrepancy.

The pulse-chase data indicate that at least a fraction of mutant N260Q gp160 is processed and that gp120 is shed from transfected cells. But what is the fate of the fraction of mutant gp160 that is not correctly processed in our assays? Because folding is not completed yet after the 4-h chase it is not unlikely that the remainder will continue to fold and leave the ER. WT gp160 already may take a full day to efficiently fold all its molecules [23], and we have not found any evidence of proteasomal degradation. We show here that at least some N260Q gp160 is degraded in the lysosomes, which will result in decreased incorporation of gp120 and g41 into the viral envelope. Hence, the eventual differences of gp120/gp41 incorporation into virions must originate from processes beyond the ER. According to Trombetta and Parodi [30], proteins with minor conformational defects can pass the quality control of the ER and move to the Golgi, where the defect is nonetheless detected. These proteins then are channelled to lysosomes for degradation. Degradation of viral proteins through lysosomes rather than proteasomes has already been described for hepatitis B virus envelope proteins [32], and according to Willey *et al.* at least uncleaved gp160 can be degraded in lysosomes [33]. The Western blot analysis revealed that mutant N260Q gp160 is cleaved to a much lesser extent than wild-type gp160. This is consistent with our findings that the effect of chloroquine on the increased gp160/gp120 (Fig. 5B) and gp41 (Fig. 5C) expression in the virus particles is more pronounced for mutant than wild-type envelope.

We have verified the hypothesis that the N260Q mutation in gp160 causes increased lysosomal degradation of gp160/gp120, by inhibiting the lysosomal degradation pathway with chloroquine, a known inhibitor of lysosomal activity. Thus, our data corroborate the hypothesis of increased lysosomal degradation due to the N260Q mutation in gp160. Virus, WT or mutant, produced in the presence of chloroquine contained increased levels of gp160, gp120 and gp41, which were clearly more pronounced for gp160 containing the N260Q mutation. Additionally, it was shown that

these virions had an increased CD4-binding potential, implying that at least part of the otherwise lysosomally degraded WT or mutant gp120 has a conformation that is competent to bind the primary cellular receptor CD4.

If the inhibition of lysosomal degradation improves gp160/gp120/gp41 incorporation in the viral envelope, we could hypothesize that virus produced in the presence of chloroquine could have an increased viral infectivity. In two independent experiments, one performed in duplo, wild-type virus derived from transfected cell cultures in the absence of chloroquine proved about 4- to 7-fold more infectious than wild-type virus produced in the presence of chloroquine (data not shown). The N260Q and N260Q/S128N mutant virus strains produced under similar experimental conditions did not show measurable infectivity in C8166 cell cultures, regardless of the presence or absence of chloroquine during virus production (data not shown). The decreased infectivity of WT virus produced in the presence of chloroquine might be due to residual chloroquine present in the virus-containing supernatant since chloroquine has been earlier shown to have a pronounced anti-HIV activity in cell culture at concentrations as low as 1–2 μM (reviewed in [34]). Furthermore, Chiang *et al.* [35] demonstrated that virus particles produced in the presence of chloroquine have a reduced viral infectivity due to interference with posttranscriptional modification of gp160 in the Golgi apparatus, resulting in decreased gp160 cleavage and deficient modification of high-mannose-type glycans into complex-type glycans. Therefore, HIV-1 production in the presence of chloroquine results in virus particles with a decreased envelope gp120 level and an increased gp160 level, resulting in an eventually decreased viral infectivity. These findings are in agreement with our results, which show an increased envelope glycoprotein (gp160+gp120) level in case of wild-type or mutant virus produced in the presence of chloroquine, and indicate that these increased envelope glycoprotein levels do not necessarily result in increased levels of viral infectivity since it is mostly uncleaved, non-functional gp160 that is incorporated, instead of functional, cleaved gp120 and gp41.

The asparagine 260 is positioned at the junction of the inner and outer domains of gp120 (Fig. 4) [36]. This region plays a crucial role in conformational changes in gp120 during viral entry into $CD4^{+}$ T-lymphocytes. The loss of the N260 glycan on gp120 may affect the native conformation of uncleaved gp160, thereby inducing misfolding sensors in the Golgi apparatus, leading to increased lysosomal degradation of the N260Q mutant glycoprotein. Mutant envelope glycoproteins that escape lysosomal degradation and are incorporated into viral particles showed a decreased CD4 binding potential, probably due to the non-optimal gp120 conformation by the lack of the N260 glycan at the junction between the inner and outer domains of gp120.

In addition to the gp120 N260 glycan which has been shown to be absolutely required for HIV infectivity in clades B and BC, Lavine *et al.* [11] and Wang *et al.* [12] showed that there are a few other gp120 N-glycans which are also crucial for viral infectivity. When comparing both reports, it is remarkable that HIV-1 strains belonging to clade CRF_07 BC seem to depend more on their N-glycans since a markedly higher number of gp120 N-glycan deletions has been shown to result in loss of viral infectivity of clade BC strains. In contrast, a limited number of gp120 N-glycan deletions was shown to be crucial for clade B infectivity. However, the gp120 N260-glycan is the only gp120 N-glycan that was shown to be indispensable for the infectivity of all studied HIV-1 strains, belonging to clades B and BC.

In conclusion, we demonstrated the absolute requirement of an intact gp120 N260 glycosylation site for the biosynthesis of HIV env glycoproteins and the infectivity of HIV-1 NL4.3 (clade B). Moreover, we demonstrated that an increased lysosomal degradation of mutant N260Q HIV env glycoproteins is involved in this loss of viral infectivity.

Acknowledgments

The authors like to thank Mrs. Cindy Heens, Leen Ingels and Els Vanstreels for excellent technical assistance, and Mrs. Christiane Callebaut for fine editorial help.

Author Contributions

Conceived and designed the experiments: LM KOF JB. Performed the experiments: LM KOF MQ. Analyzed the data: LM KOF MQ IB. Contributed to the writing of the manuscript: LM KOF MQ IB JB.

References

1. Hu Q, Mahmood N, Shattock RJ (2007) High-mannose-specific deglycosylation of HIV-1 gp120 induced by resistance to cyanovirin-N and the impact on antibody neutralization. Virology 368: 145–154.
2. Li Y, Cleveland B, Klots I, Travis B, Richardson BA, et al. (2008) Removal of a single N-linked glycan in human immunodeficiency virus type 1 gp120 results in an enhanced ability to induce neutralizing antibody responses. J Virol 82: 638–651.
3. Reitter JN, Means RE, Desrosiers RC (1998) A role for carbohydrates in immune evasion in AIDS. Nat Med 4: 679–684.
4. Jitsuhara Y, Toyoda T, Itai T, Yamaguchi H (2002) Chaperone-like functions of high-mannose type and complex-type N-glycans and their molecular basis. J Biochem 132: 803–811.
5. H, Holschbach C, Hunsmann G, Schneider J (1988) Carbohydrates of human immunodeficiency virus. Structures of oligosaccharides linked to the envelope glycoprotein 120. J Biol Chem 263: 11760–11767.
6. CK, Spellman MW, Riddle L, Harris RJ, Thomas JN, et al. (1990) Assignment of intrachain disulfide bonds and characterization of potential glycosylation sites of the type 1 recombinant human immunodeficiency virus envelope glycoprotein (gp120) expressed in Chinese hamster ovary cells. J Biol Chem 265: 10373–10382.
7. C, Doores KJ, Dunlop DC, Thaney V, Dwek RA, et al. (2011) The glycan shield of HIV is predominantly oligomannose independently of production system or viral clade. PLoS One 6: e23521.
8. KJ, Bonomelli C, Harvey DJ, Vasiljevic S, Dwek RA, et al. (2010) Envelope glycans of immunodeficiency virions are almost entirely oligomannose antigens. Proc Natl Acad Sci U S A 107: 13800–13805.
9. WR, Syu WJ, Du B, Matsuda M, Tan S, et al. (1992) Nonrandom distribution of gp120 N-linked glycosylation sites important for infectivity of human immunodeficiency virus type 1. Proc Natl Acad Sci U S A 89: 2213–2217.
10. RL, Smith DH, Lasky LA, Theodore TS, Earl PL, et al. (1988) In vitro mutagenesis identifies a region within the envelope gene of the human immunodeficiency virus that is critical for infectivity. J Virol 62: 139–147.
11. CL, Lao S, Montefiori DC, Haynes BF, Sodroski JG, et al. (2012) High-mannose glycan-dependent epitopes are frequently targeted in broad neutralizing antibody responses during human immunodeficiency virus type 1 infection. J Virol 86: 2153–2164.
12. W, Nie J, Prochnow C, Truong C, Jia Z, et al. (2013) A systematic study of the N-glycosylation sites of HIV-1 envelope protein on infectivity and antibody-mediated neutralization. Retrovirology 10: 14.
13. KO, Balzarini J (2011) The highly conserved glycan at asparagine 260 of HIV-1 gp120 is indispensable for viral entry. J Biol Chem 286: 42900–42910.
14. J, Weberova J, Carobene M, Mirza M, Martinez-Picado J, et al. (2006) Use of a novel assay based on intact recombinant viruses expressing green (EGFP) or red (DsRed2) fluorescent proteins to examine the contribution of pol and env genes to overall HIV-1 replicative fitness. J Virol Methods 136: 102–117.
15. K, Dekeersmaeker N, Schrooten Y, Weber J, Schols D, et al. (2009) Novel recombinant virus assay for measuring susceptibility of human immunodeficiency virus type 1 group M subtypes to clinically approved drugs. J Clin Microbiol 47: 2232–2242.
16. BS, Thayer RM, Vincent KA, Haigwood NL (1991) Effect of intron A from human cytomegalovirus (Towne) immediate-early gene on heterologous expression in mammalian cells. Nucleic Acids Res 19: 3979–3986.

17. V, Cherepanov P, Van Laethem K, Hantson A, Van Remoortel B, et al. (2002) env chimeric virus technology for evaluating human immunodeficiency virus susceptibility to entry inhibitors. Antimicrob Agents Chemother 46: 3954–3962.
18. J, Desai K, Darbyshire J (2005) The development of vaginal microbicides for the prevention of HIV transmission. PLoS Med 2: e142.
19. Laethem K, Schrooten Y, Lemey P, Van Wijngaerden E, De Wit S, et al. (2005) A genotypic resistance assay for the detection of drug resistance in the human immunodeficiency virus type 1 envelope gene. J Virol Methods 123: 25–34.
20. J, Francois KO, Covens K, Van Laethem K, Balzarini J (2008) Glycan deletions in the HIV-1 gp120 V1/V2 domain compromise viral infectivity, sensitize the mutant virus strains to carbohydrate-binding agents and represent a specific target for therapeutic intervention. Virology 382: 10–19.
21. RW, Busser E, Moore JP, Lu M, Berkhout B (2004) Evolutionary repair of HIV type 1 gp41 with a kink in the N-terminal helix leads to restoration of the six-helix bundle structure. AIDS Res Hum Retroviruses 20: 742–749.
22. RW, Dankers MM, Busser E, Caffrey M, Moore JP, et al. (2004) Evolution of the HIV-1 envelope glycoproteins with a disulfide bond between gp120 and gp41. Retrovirology 1: 3.
23. A, Zonneveld D, Braakman I (2003) Folding of HIV-1 envelope glycoprotein involves extensive isomerization of disulfide bonds and conformation-dependent leader peptide cleavage. FASEB J 17: 1058–1067.
24. UK, Molbert E, Showe M, Kellenberger E (1970) Form-determining function of the genes required for the assembly of the head of bacteriophage T4. J Mol Biol 49: 99–113.
25. A, Aebi M (2001) Intracellular functions of N-linked glycans. Science 291: 2364–2369.
26. I, Land A, Eggink D, Verkade E, Tuin K, et al. (2009) Optimization of human immunodeficiency virus type 1 envelope glycoproteins with V1/V2 deleted, using virus evolution. J Virol 83: 368–383.
27. RW, Hsu ST, van Anken E, Liscaljet IM, Dankers M, et al. (2008) Evolution rescues folding of human immunodeficiency virus-1 envelope glycoprotein GP120 lacking a conserved disulfide bond. Mol Biol Cell 19: 4707–4716.
28. RL, Ross EK, Buckler-White AJ, Theodore TS, Martin MA (1989) Functional interaction of constant and variable domains of human immunodeficiency virus type 1 gp120. J Virol 63: 3595–3600.
29. Y, Bergeron JJ, Luo L, Ou WJ, Thomas DY, et al. (1996) Effects of inefficient cleavage of the signal sequence of HIV-1 gp 120 on its association with calnexin, folding, and intracellular transport. Proc Natl Acad Sci U S A 93: 9606–9611.
30. ES, Parodi AJ (2003) Quality control and protein folding in the secretory pathway. Annu Rev Cell Dev Biol 19: 649–676.
31. JP, Willey RL, Lewis GK, Robinson J, Sodroski J (1994) Immunological evidence for interactions between the first, second, and fifth conserved domains of the gp120 surface glycoprotein of human immunodeficiency virus type 1. J Virol 68: 6836–6847.
32. C, Macovei A, Petrescu S, Branza-Nichita N (2012) Activation of ERAD pathway by human hepatitis B virus modulates viral and subviral particle production. PLoS One 7: e34169.
33. RL, Bonifacino JS, Potts BJ, Martin MA, Klausner RD (1988) Biosynthesis, cleavage, and degradation of the human immunodeficiency virus 1 envelope glycoprotein gp160. Proc Natl Acad Sci U S A 85: 9580–9584.
34. A, Gennero L, Sperber K, Boelaert JR (2001) The anti-HIV-1 activity of chloroquine. J Clin Virol 20: 131–135.
35. G, Sassaroli M, Louie M, Chen H, Stecher VJ, et al. (1996) Inhibition of HIV-1 replication by hydroxychloroquine: mechanism of action and comparison with zidovudine. Clin Ther 18: 1080–1092.
36. PD, Wyatt R, Robinson J, Sweet RW, Sodroski J, et al. (1998) Structure of an HIV gp120 envelope glycoprotein in complex with the CD4 receptor and a neutralizing human antibody. Nature 393: 648–659.
